# Multi-sectoral Approach to Non-Communicable Diseases Control: Easier Said than Done

**DOI:** 10.31729/jnma.4729

**Published:** 2019-12-31

**Authors:** Kishori Mahat, Badri Thapa

**Affiliations:** 1 Non-Communicable Disease Unit, The Nossal Institute of Global Health, School of Population and Global Health, University of Melbourne, Melbourne, Australia; 2 Public Health Specialist, Bishalnagar, Kathmandu, Nepal

**Keywords:** *multi-sectoral*, *non-communicable diseases*, *risk factors*, *sustainable development goals*

## Abstract

The burden of non-communicable diseases is growing and countries are committed to combat this and achieve the sustainable development goals and targets. Non-communicable diseases are complex conditions attributed by multiple behavioural risk factors and without understanding the whole ecosystem of such diseases, it is difficult to determine the global goals and targets for them and to take action to address them. Countries are trying to take the multi-sectoral approach in addressing the non-communicable diseases and often encounter challenges in operationalizing the approach. Therefore, it is essential to nuance the multi-sectoral approach to non-communicable diseases in order to better inform application to achieving the sustainable development goals for which multisectoral approach is imperative.

## INTRODUCTION

Non-Communicable Diseases (NCDs) are responsible for about two-thirds of deaths worldwide, mostly in Low and Middle-Income Countries (LMICs).

UN Inter-Agency and Experts Group on the Sustainable Development Goals (IAED-SDGs) have proposed changes to 15 out of the 17 SDGs in which changes to the health specific SDG 3 is also included.^1^ The SDG 3.4 is to ‘reduce by one third premature mortality from NCDs through prevention and treatment and promote mental health and well-being by 2030.’

The aim of this viewpoint is to improve the understanding of the ‘complex’ nature of the NCDs and the multisectoral approach so that relevant action can be taken to reduce its burden.

### WHAT MAKES NCDs COMPLEX?

The NCDs are complex because these conditions are attributed to multiple risk factors. The social determinants,^2^ meaning the conditions in which people are born, grow, live, work, and age shape the distribution of the four main behavioural risk factors. These are the unhealthy diet, physical inactivity, tobacco smoking, and excess alcohol consumption. Taking an example of one risk factors like alcohol, the association between the societal factors, cultural norms, neighbourhoods, and social contexts with alcohol misuse is well established^3^ and the evidence based on influence of other risk factors and NCDs are well established.

These behavioural risk factors often also trigger stress pathways affecting mental health and other NCDs. Stress is also associated with unhealthy behaviours that are risk for NCDs. Further, the environmental exposure to pollutants is linked to specific NCDs. The social determinants on the other hand also influence health seeking behaviour, prevention, diagnosis, and treatment practices. Thus, managing NCDs is certainly complex and calls for multi-sectoral approach to prevent NCDs by reducing their major risk factors.^4^

### NUANCES OF THE MULTI-SECTORAL APPROACH

The multi-sectoral approach refers to actions that are undertaken by sectors outside the health sector, with or without the collaboration with the health sector to attain health-related outcomes or influence health determinants.^5^ In recent days, the concept of life course approach to prevention and control of the NCDs is also becoming popular, as it can help determine when and how to influence the social determinants of health.^6^ But, even taking the life course approach can be challenging as it requires multi-sectoral actions extending beyond the health sector and that is targeted within the natural settings that people encounter through the various stages of their lives.^6^

The multi-sectoral approach recognizes the fact that the social and economic factors influencing population health do not only lie inside the health sector but are also found within other sectors.^7^

Within the spirit of the multi-sectoral approach, WHO has been encouraging countries to develop the multisectoral action plans to address NCDs, but the progress has been sluggish with only 41 percent of the countries with multi-sectoral action plans for NCDs.^8^ Among those action plans developed, the operationalization is limited to only 34 percent.^8^ Often multi-sectoral approach is limited to the formation of high-level steering committees with representation from different ministries, which are rarely functional.

Although, the multi-sectoral approach can help the groups with different interests stimulate a more robust sense of institutional legitimacy and take more unified action to address health priorities,^7^ operationalizing this has been challenging. Evidence from Africa indicates that the major barriers to multi-sectoral action is the lack of awareness by various sectors about their potential contribution, roles, weak political will, coordination complexity and inadequate resources.^7^ These barriers of multi-sectoral approach to NCDs holds true for any public health programs requiring the multi-sectoral approaches like combating antimicrobial resistance, malnutrition and diseases impacted by climate change e.g. vector borne diseases programs. The challenge to countries then is a continuous juggle among the multiple ‘multi-sectoral committees’ formed to oversee different programs requiring multi-sectoral approach.

Many studies also highlight the importance of taking the ‘whole systems’ approach to reduce the burden of NCDs. This approach integrates action on the social determinants of health and action across all major areas of society that influence health.^9^ But the real-world implementation of this requires first, understanding the whole systems in the context of NCDs and second how to navigate through the whole system to foster the multi-sectoral approach, should this be the best approach forward. The question then is, ‘Are Ministries of Health (MoH) well suited to lead the multi-sectoral initiatives?’ With only the technical capacity on NCDs management and less control on financial resources of its own, the role of MoH in driving the multi-sectoral approach for NCDs and similar initiatives seems to be limited. This is a pertinent issue to be addressed if countries are committed towards achieving the SDGs^10^ which requires the multi-sectoral approach.

### WAY FORWARD

The learnings from implementing the multisectoral approach to NCDs will not only benefit in demystifying the multi-sectoral approach to NCDs but will also inform application of this approach in achieving other SDGs. To realise the multi-sectoral approach to NCDs in its real sense, below are some of the recommendations;

- The political commitment and leadership are the most important factors for successful implementation of the multi-sectoral coordination. Often political commitment is limited to being signatories to the global goals and are not translated into action by allocation of resources required to achieve the goals like the financial and human resources which has been one of the bottlenecks for NCDs programs. A technical lead agency for NCDs like WHO allocated less than 5 percent of its total biennial budget to NCDs in 2016-17 which further decreased in 2018-2019.^8^ Therefore, allocation of resource for NCDs by national governments and supporting organizations is essential.
- Prevention and management of NCDs requires the whole-of government and whole-of systems approach with effective role played by sectors beyond health.^11^ This requires establishing clear roles and responsibilities of each sector vis-à-vis NCDs with establishing coordinating body or board above all ministries. This will help minimize the power conflict which often creeps in when health sector coordinates such initiatives with other ministries with equal footing.
- Studies have shown that the network of NCDs experts are still dominated by the health sector and has not successfully expanded to other stakeholders whose engagement is required.^12^ Therefore, identifying the added advantage of the health sector over other sectors in prevention and control of NCDs is required rather than health sector trying to do everything.
- Since multi-sectoral approach is key to achieving SDGs, establishing an institutional mechanism with stringent monitoring and evaluation and regular coordination and communication is required, else running ‘multiple’ multi-sectoral committees in parallel with different program focus and silo reporting mechanisms will be very chaotic for countries to manage.
- Often, successful interventions are flaunted and published and seldom are the failures. But there is so much that could be learnt from the failed initiatives as the saying goes, ‘to err is human.’ So, establishing the culture of harnessing the failures for continuous learning is required.
- It is time to think out of the box. Currently, primary health-care services in many countries including Nepal do not have the capacity to diagnose or manage NCDs. Therefore, reorienting the health system from its traditional approach to manage communicable diseases, to the management of NCDs is needed. The Ministry of Health is best suited to advocate on this but needs support from other key ministries like Ministry of Finance and other stakeholders based on country context.
- In implementing the multi-sectoral approach, it is essential to be mindful of the fact that there could be lack of common knowledge and understanding of different programs among various stakeholders. While appreciating the diversity in knowledge and expertise that comes with the multi-sectoral approach, timely information dissemination and regular communication are essential to promote informed decision making.
- Evidence generation in the critical and emerging areas of NCDs including cross-sectoral benefits are essential. The combination of increasing prevalence of NCDs in LMICs and increasing lifespans in many countries may be leading to a changing spectrum of the types of morbidity that accompany NCDs.^13^ But there is little evidence to inform this. Therefore, investment in research to understand the life course approach to various NCDs and identifying which sector can play what role and when is essential.

## Conflict of Interest

**None.**

## References

[1] United Nations. IAEG-SDGs 2020 Comprehensive Review (2019). [Internet].

[2] World Health Organization: Social determinants of health (2019). [Internet].

[3] Sudhinaraset M, Wigglesworth C, Takeuchi DT (2016). Social and Cultural Contexts of Alcohol Use: Influences in a Social-Ecological Framework. Alcohol Res.

[4] Ezzati M, Riboli E (2012). Can noncommunicable diseases be prevented? Lessons from studies of populations and individuals. Science.

[5] Arora M, Chauhan K, John S, Mukhopadhyay A (2011). Multi-Sectoral action for addressing social determinants of noncommunicable diseases and mainstreaming health promotion in National Health Programmes in India. Indian J Community Med.

[6] Mikkelsen B, Williams J, Rakovac I, Wickramasinghe K, Hennis A, Shin HR (2019). Life course approach to prevention and control of non-communicable diseases. BMJ.

[7] Juma PA, Mohamed SF, Matanje Mwagomba BL, Ndinda C, Mapa-tassou C, Oluwasanu M (2018). Non-communicable disease prevention policy process in five African countries authors. BMC Public Health.

[8] Alwan A (2017). The NCD Challenge: Progress in responding to the global NCD challenge and the way forward.

[9] Marmot M, Bell R (2019). Social determinants and non-communicable diseases: time for integrated action. BMJ.

[10] United Nations (2019). Sustainable development Goal 3 Ensure healthy lives and promote well-being for all at all ages.

[11] Saha A, Alleyne G (2018). Recognizing noncommunicable diseases as a global health security threat. Bull World Health Organ.

[12] Heller O, Somerville C, Suggs LS, Lachat S, Piper J, Piper J (2019). The process of prioritization of non-communicable diseases in the global health policy arena. Health Policy and Planning. Health Policy Plan.

[13] Wright AK, Kontopantelis E, Emsley R, Buchan I, Sattar N, Rutter MK (2017). Life Expectancy and Cause-Specific Mortality in Type 2 Diabetes: A Population-Based Cohort Study Quantifying Relationships in Ethnic Subgroups. Diabetes Care.

